# Trauma and Pain in Family-Orientated Societies

**DOI:** 10.3390/ijerph15010044

**Published:** 2017-12-28

**Authors:** Jan Ilhan Kizilhan

**Affiliations:** 1 Mental Health and Addiction, Co-operative State University Baden-Württemberg, Schramberger Str. 26, 78054 Villingen-Schwenningen, Germany; kizilhan@dhbw-vs.de; 2 Institute for Psychotherapy and Psychotraumatology, University of Duhok, 42001 Duhok, Irak; 3 Department of Transcultural Psychosomatic, MediClin-Klinik am Vogelsang Donaueschingen, 78166 Donaueschingen, Germany

**Keywords:** post-traumatic stress disorder (PTSD), pain, pain perception, understanding of illness, culture, family-oriented societies

## Abstract

People from family-oriented societies in particular, in addition to having a post-traumatic stress disorder (PTSD) suffer from chronic pain and physical complaints. Such people have a different understanding of physical illness and pain and, compared to patients from western societies, have different ideas on healing, even when confronted with the therapist. Hitherto, these factors have not been sufficiently taken into account in modern, multi-module therapy approaches. Trauma can be perceived via pain and physical complaints, whereby the pain is not restricted to one part of the body but is seen as covering the body as a whole. Therefore, in the treatment and above all in the patient-therapist relationship, it is necessary to understand what importance is attached to the perceived pain in relation to the trauma. The afflicted body expresses the trauma in the shape of its further-reaching consequences such as the patient’s social, collective, economic and cultural sensitivity. Therefore, for the effective treatment of trauma and chronic pain, it is necessary to use a multi-modal, interdisciplinary, and culture-sensitive approach when treating patients from traditional cultural backgrounds.

## 1. Introduction

As a result of the increasing numbers of people from other cultures who flee and migrate for political, religious or economic reasons or because of war—in recent years, mainly refugees from Asia and Africa—doctors and therapists are reporting that traumatized refugees from family-oriented societies—such as Syria, Iraq, or Afghanistan—in addition to mental complaints caused by traumatization, complain of pain more than patients from the western world [1,2]. They believe that psychotherapy is not very suitable on its own and think that medication or even an operation would be the better course of treatment [3]. In addition to flashbacks, intrusions, fears, etc. total body pain is at the forefront and this indicates a different perception and processing of traumatic experiences [4].

Unlike the western society, which puts impetus on ‘individualism’, the traditional family oriented society is ‘collectivistic’ in that it promotes interdependence and co-operation, with the family forming the focal point of this social structure. The traditional family oriented society families like in Syria, Iraq, Afghanistan and some part of south European countries are therefore far more involved in caring of its members, and also suffer greater illness burden than their western counterparts.

In family-oriented societies the mention of psychotherapy can give rise to the feeling that they are in some way ‘mad’ and that they are ostracized and that medication or ‘resting the body’ can reduce the pain. A further reason for rejecting psychotherapy is the collective thinking in which the family plays a superordinate role. For this reason, personal feelings and inner mental symptoms are often not expressed; to adapt to the social environment is considered a sign of personal maturity [5]. The human body is ‘allowed’ one illness however, which is why both physical and mental complaints are expressed via the body. On examination, therefore, it is initially relatively difficult to establish whether the patient is suffering from a physical or a mental illness or both.

The literature describes the treatment and relationship structure of patients with PTSD and a pain disorder from family-oriented societies as difficult [1,4,6] and therefore this article will offer an overview of the understanding of trauma and pain perception and the cultural differences surrounding trauma and pain disorders and will outline some possible new treatment strategies.

## 2. Culture, Trauma, and Pain

The diagnostics of mental disorders and the consequences of PTSD orient towards the criteria of ICD-10 and DSM-V. This assumes that all people display comparable stress and reactions following a traumatic experience. This is, however, not substantiated by clinical experience and the findings of transcultural psychiatry [7].

The link between PTSD and chronic pain has been proven in numerous studies. In a study with general psychiatric outpatients Villano and colleagues [8] found that 46% of those examined fulfilled the criteria of a PTSD, 40% reported chronic pain, and 24% of the patients were diagnosed with both. The results of an examination of war veterans showed a comorbidity rate for PTSD and chronic pain of 66% [9]. Other studies with war veterans showed that, in parts, well over 80% of those examined fulfilled the criteria for both disorders [10]. The data of a very comprehensive Canadian random test with 36,984 test persons (Canadian Community Health Survey Cycle) show a considerable discrepancy with regard to chronic pain between people with and without a diagnosed PTSD: of the PTSD patients, 46% suffered from chronic back pain (compared to 20.6% of those examined who did not have PTSD), and 33% suffered from migraine (compared to 10%) [11].

According to Otis and colleagues [9] the prevalence of pain among PTSD patients is 34–80% significantly higher than the other way round, where the PTSD prevalence among pain patients is 10–50%. This imbalance can be explained in part by the fact that traumatic experiences are often linked to physical pain and that these represent a kind of post-traumatic disorder [4].

A study by Norman and colleagues [12] with patients at a trauma centre at the University of California showed that pain immediately after the traumatic incident was a risk factor for developing PTSD. According to the authors, the link may be the result of a more negative assessment of the trauma recollection as a result of the pain and increased stress associated with the trauma. In comparison to German women who had been victims of sexualized violence, female victims of sexualized violence from Turkey complained more about physical pain and somatic ailments than the German women and in some cases developed a cleansing compulsion [13].

The literature indicates a high degree of co-occurrence between pain and PTSD, regardless of whether the pain is being assessed in patients with PTSD or PTSD is being assessed in patients with chronic pain. Also, they may interact in such a way as to negatively affect the course and outcome of treatment of either disorder [9]. The high comorbidity between these disorders has been postulated as being due to either shared vulnerability or mutual maintenance [14,15].

Every culture has developed its own management strategies appropriate to their own values and norms to cope with physical ailments in connection with pain. The Irish, for example, shrank rather from any contact with others because they thought it was indelicate to show pain [16]. North Americans went the doctor’s as early as possible and described their symptoms without showing any emotion so that the doctor could immediately initiate a rational treatment [16]. Southern Europeans expressed their pain loudly and clearly so that those around them could sympathize. Filipinos acquiesced to their fate in a fatalistic manner [16].

Psychological problems following trauma manifest themselves, amongst other things, in the form of physical disorders appropriate to the person’s cultural imprint [17]. Nigerians, when they report fear and depression, talk of “a sensation of heat” in their head, “writhing maggots”, as well as a “biting sensation” in their whole body [18]. In psychiatric clinics in China they report “weak nerves” along with fatigue, headache, vertigo, and gastrointestinal disorders [17,19]. Many people from South America and the Mediterranean react to psychological burdens with headaches and muscle pain, heat sensations and pins and needles in the feet, heart problems, and stomach complaints [18]. In some parts of India and the Middle East, rheumatic and rheumatoid pains are described as “wind” pains. Also reported are “wandering” pains which manifest themselves in a different part of the body each day [16].

Patients from family-orientated societies, predominantly from rural Turkey, Iraq, or Syria, report pain disorders significantly more often than western patients and this explains the high number of pain diagnoses [6]. Those affected speak of all complaints as if they were physical, i.e., the sufferers seem to be stuck in an archaic idea of illness. Subjective suffering can be expressed symbolically with fatigue, crying, walking with aids, etc. The patients present themselves as broken and weak people. As a rule, they remain consistent to this regressive and appellative stance so that they remain inactive even in their home environment.

This severe fixation with total-body pains, the cause of which is often difficult to elicit, frequently leads to problems of diagnosis and treatment [16].

## 3. Diagnostics

Fundamentally, the concepts of PTSD and cognitive behavioral therapy are applicable to all ethnic groups. However, the varying perceptions of health and disease and the cultural, traditional, medical treatment for dealing with traumatic experiences require alternative concepts or additions.

The culture-specific aspects of patients from traditional cultures are their knowledge of the anatomy and physiology of their own body and their concept of pain (seen as magic, a curse, punishment, etc.). The experiencing of pain is not limited to one part of the body but is seen holistically i.e., over the body as a whole. Therefore, even when initially documenting the pain—i.e., localization, quality, time pattern, and cause—the patients give other details than those to be expected from northern European patients [4]. For example, ethnic Turkish patients, in contrast to German ones, see fate or extreme environmental factors as responsible for their illness. In addition to their war traumatization, some refugees are so bitter that they try to escape the injustice they feel as a kind of regression through physical pain [4,20]. The way in which people report their pain or in some cases do not speak about it has an important influence on how the therapist takes down their case history and consequently correctly diagnoses the complaints.

Understanding illness is especially important, both for the diagnosis and for the treatment. The comparative study on ethnic Turkish and German patients in a psychosomatic clinic revealed that significantly fewer ethnic Turkish patients were able to describe their illness [7]. It is necessary to analyze the circumstances of the onset or aggravation of the pain and to consider the individual and collective biography in doing so (e.g., ostracism because of the ethnic and/or religious denomination in their country of birth, migration, culture and generation conflicts, etc.) in order to elucidate the indications for the triggering factors. The aim is to find out what diminishes or intensifies the pain and what restrictions arise as a result [20].

However, this pain patient must feel that his complaints and ailments are being taken seriously; otherwise he will stop the therapy [8]. In general, patients from traditional societies do not present their problems very chronologically so that what they say, viewed initially superficially, does not seem to relate clearly to their complaint.

The aim of psychological pain therapy is to reduce the patient’s impairment since this normally leads to a reduction in the subjective pain intensity. Since patients from traditional societies have a pain model reduced to somatic variables and a specific understanding of anatomy, the treatment setting has to be somewhat modified for them. Their causal and control attributions are determined in part, for example, by simple biomedical assumptions originating in the Arabic—Greek medicine of the “Four Humours Theory” and magical connotations [20]. The way in which patients from family-oriented societies present their complaints together with their different ideas of anatomy and its functions with respect to mental illnesses are to be considered when diagnosing. Consequently, a suitable treatment and explanation model must be developed with the patient, which is appropriate to their level of education and cultural perceptions [16].

## 4. Treatment

On the whole, in the case of a large number of patients from family-oriented societies the activity spectrum appears to be significantly limited. As a result, their life revolves around the pain; it becomes the focus of their thinking and behavior [16,19]. Their assumption that the body has to rest when it experiences pain leads to a passive relieving posture. It therefore presents a great challenge if we wish to increase the room for manoeuvre necessary to counteract the patient’s restrictions: behavioral (little movement), emotional (reduction of depression and helplessness) and cognitive (limitation to pain). Breaking down avoidance behavior is therefore a high priority when carrying out the therapy.

Owing to the various ways pain is presented, the passive relieving posture, language problems, and possible ethnic-cultural behavioral differences, it is important to form a relationship based on trust with the patient. With patients from family-oriented societies the physician (the clinical psychologist too, is regarded as a physician/“doctor”) is traditionally seen as a father-like family friend [20]. He represents a figure of authority who cultivates an active, knowing, and advisory role with the patient and his family. He must accept this cultural role if he does not wish to considerably unsettle the patient.

Whereas with a German patient the main focus is on mobilising the individual’s potential, the patients mentioned above expect more help from the person in authority and this must be offered [21]. This means, however, that the therapist must develop an awareness of his own cultural attachment and should be able, from this position, via his (counter-) transpositions (individual and social prejudices and stereotypes) to “de-actualize” his attitudes and avoid presenting them to the patient before they affect the therapy in a destructive way. Only then will the patient be willing to alter his behavior at a mental and physical level [19].

The traditional exposition therapy is not always effective with victims of political oppression and with people suffering from complex and cumulative traumatization [20,21]. It can even be counter-productive and reduce compliance, increase pain perception and also the drop-out rate. There are reports that not all, but some, patients find it more helpful, as a first step, to concentrate on the pain and its treatment rather than dealing directly with the traumatizing incidents. It is also necessary to discuss whether repression and avoidance, in other words keeping up the pain, would not present a better coping strategy [7]. In some cultures, living with pain and suppressing the trauma is regarded as a successful coping mechanism. This is particularly prevalent in collective societies in which social harmony is the highest priority [22]. Here, in particular, the healing process is determined by the cultural and social context, and care is taken to make sure that the victim does not ‘lose face’. This applies especially to politically-motivated violence [23]. A conversation with these patients on the topic of stress is normally avoided.

### Case Casuistry

Yasemin is a Yazidi and her family were persecuted and taken hostage by the “Islamic State” (ISIL). She was repeatedly raped and sold before she was able to escape from the hands of her tormentors. She reports how the IS fighters took her prisoner; she talks of violence, rape, escape, and unimaginable suffering. She says she was sold 12 times to IS fighters in Iraq and Syria, and beaten and raped again and again.

Finally, after eight months of captivity, she managed to flee from Syria. She says more than 20 members of her family were murdered by the ISIL. She had to watch her husband being executed. She is nervous, desperate and shows me a bag full of pain killers and sleeping pills because she has pain all over her body and cannot sleep. “If only I didn’t have this pain, I would be much better. The doctors say my body is all right. The pains are due to my mental state. I don’t understand that. I am in pain, I can feel it all over my body.” As regards the pain, she has the feeling that her body is besmirched. She feels she has to shower several times every day and yet she still has the feeling that her body is unclean.

Just how far psychotherapeutic trauma work is possible seems also to depend on how a society deals with the topic of sexualized violence. In this context, patients often report of a considerable feeling of insecurity, not to say viewing the topic as an absolute taboo. High moral ideas and restrictions, especially in women, lead to considerable worry and fear since they are particularly at risk of being ostracized by the collective. In this respect, feelings of shame play a special part. This is due to the fact that, in a so-called shame culture, the actual event and the violation of a norm are of less importance than the desire to keep one’s face. For example, the collective may regard the rape of a young woman as shameful and the victim ostracized. The fact that the perpetrator is also seen as violating a norm is of secondary importance in the collective.

From the psychodynamic perspective, discussing traumatic incidents via *statements of pain* [24] offers people with severe traumatic experiences the possibility of transferring the ostracism, social affront, feelings of guilt and inferiority away from their conscious experience and on to a physical level. In this way they maintain their self-esteem and at the same time hope that the doctor and medicine can help them [7,11].

In addition to feelings of mental and physical tension, the perception of pain is also influenced by increased inactivity and avoidance behavior. As a result, the fear of possible consequences, so-called “catastrophe thinking” and “fear avoidance beliefs” the cycle of pain results in an increase of inactivity, avoidance behavior and depression.

The crucial link between the PTSD and the pain chain is avoidance/inactivity and the associated depression. These factors have a crucial influence on the development and maintenance of both the PTSD and chronic pain [25,26].

Following the diagnostics, a further treatment model is psycho-education as a necessary integral part of the therapy. Psycho-education is designed to reinforce the patient’s own self-efficacy beliefs. This can be in the form of media such as self-help brochures and videos in the patient’s mother tongue. One essential target of this education is to demonstrate to the patient that physical and mental processes are intertwined. In doing so, the culture-specific aspects and the patient’s level of education should be taken into account [27].

## 5. Conclusions

A culture-sensitive approach to the treatment of traumatized individuals with chronic pain and physical ailments from family-oriented societies is indicated when the patient is severely restricted and needs medical help; when psychological factors influence his perception of pain and this impairment can be verified in the diagnosis; when conventional treatment does not work sufficiently well on the patient due to his different understanding of illness and how to cope with trauma and pain.

An individual and culture-sensitive treatment which takes the relationship structure between the patients and therapist into consideration is especially important, whereby traumatologists and doctors co-operate with other professional groups (sport therapists, physiotherapists, creative therapists, etc.) and look at the patient’s state of the art cultural imprint [3,17].

If language, cultural, and migration-specific aspects are included in the consultation, treatment and social support of patients from family-oriented societies with PTSD and chronic pain, it is possible to fundamentally improve their care and integration [28]. Therefore, on the part of both the therapist and the health institutes, specific transcultural knowledge and the consideration of the social and political structures of the health institutes are necessary to be able to treat these patients early enough and adequately and in this way, for instance, to prevent a chronification of the illness [24]. In addition to multicultural teams of therapists, it is above all necessary to make all staff aware of the need to take a transcultural, culturally-sensitive perspective [29,30].

Treating patients with PTSD and chronic pain from family-oriented societies is not about learning a new form of psychotherapy. It is about registering and learning skills of the culture-sensitive use of psychotherapeutic treatment in general and especially in behavioral trauma therapy and chronical pain methods [24]. Individual therapy is also about concentrating on people from different cultures, with a different concept of illness and how to deal with it [1,2,31]. This requires being willing to reflect and possessing a critical attitude to one’s own work whilst at the same time remaining impartial and open to the patients’ concerns. Transcultural competence is needed and means that it is necessary to reflect on one’s own culture in order to understand other cultures [17,31].

When dealing with patients from family-oriented societies with PTSD and chronic pain it is necessary to consider cultural, historical (trauma) and socio-political aspects, the perception of illness and dealing with it and the way in which a relationship can be formed with the patient [21,25]. In addition, alternative therapy approaches are important, involving an interdisciplinary and culturally-sensitive focus in the psychiatrists and psychotherapists, as is close co-operation with other professional groups and the patient’s state-of-the-art cultural imprint.

## References

[1] Hassan G., Ventvoegel P., Jeffee-Bahloul H. (2016). Mental health and psychosocial wellbeing of Syrians affected by armed conflict. Epidemiol. Psychiatr. Sci..

[2] Hinton D., Kirmayer L.J. (2016). The flexibility hypothesis of healing. Cult. Med. Psychiatry.

[3] Kizilhan J.I. (2011). Zum psychotherapeutischen arbeiten mit migrantinnen und migranten in psychosomatisch-psychiatrischen kliniken. Psychotherapeutenjournal.

[4] Kizilhan J.I. (2012). Kultursensible Psychotherapie.

[5] Heine P., Assion H.J., Assion J. (2005). Traditionelle Medizin in islamischen Kulturen. Migration und Seelische Gesundheit.

[6] Schiltenwolf M., Pogatzki-Zahn E.M. (2005). Schmerzmedizin aus einer interkulturellen und geschlechterspezifischen Perspektive. Schmerz.

[7] Schouler-Ocak M. (2015). Trauma and Migration: Cultural Factors in the Diagnosis and Treatment of Traumatised Immigrants.

[8] Villano C.L., Rosenblum A., Magura S. (2007). Prevalence and correlates of posttraumatic stress disorder and chronic severe pain in psychiatric outpatients. J. Rehabil. Res. Dev..

[9] Otis J.D., Keane T.M., Kerns R.D. (2003). An examination of the relationship between chronic pain and posttraumatic stress disorder. J. Rehabil. Res. Dev..

[10] Shipherd J.C., Keyes M., Jovanovic T., Ready D.J., Baltzell D., Worley V., Gordon-Brown V., Hayslett C., Duncan E. (2007). Veterans seeking treatment for posttraumatic stress disorder: What about comorbid chronic pain?. J. Rehabil. Res. Dev..

[11] Broeckman B.F.P., Olff M., Boer F. (2007). The genetic background to PTSD—Review. Neurosci. Biobehav. Rev..

[12] Dunmore E., Clark D.M., Ehlers A. (2001). A prospective investigation of the role of cognitive factors in persistent posttraumatic stress disorder (PTSD) after physical or sexual assault. Behav. Res. Ther..

[13] Ehlers A., Clark D.M. (2000). A cognitive model of persistent posttraumatic stress disorder. Behav. Res. Ther..

[14] Kizilhan J.I. (2010). Interaktion von trauma und reinigungszwang und religion bei patientinnen mit einer PTSD. Eine vergleichende studie. Verhaltensmed. Verhaltensther..

[15] Asmundson G.J.G., Coons M.J., Taylor S., Katz J. (2002). PTSD and the experience of pain: Research and clinical implications of shared vulnerability and mutual maintenance models. Can. J. Psychiatry.

[16] Sharp T.J., Harvey A.G. (2001). Chronic pain and posttraumatic stress disorder: Mutual maintenance?. Clin. Psychol. Rev..

[17] Kizilhan J.I. (2017). Patient form middle east and the impact of culture on psychological pain-treatment. Fibrom Open Access.

[18] Edigbo P. (1986). A cross sectional study of somatic complaints of Nigerian females using the Enugu Somatization Scale. Cult. Med. Psychiatry.

[19] Poundja J., Fikretoglu D., Brunet A. (2006). The co-occurence of posttraumatic stress disorder symptoms and pain: Is depression a mediator?. J. Trauma. Stress.

[20] Gatchel R., Peng Y., Peters M., Fuchs P., Turk D. (2007). The biopsychosocial approach to chronic pain: Scientific advances and future direction. Psychol. Bull..

[21] Kizilhan J.I. (2016). Die deutung des schmerzes in anderen Kulturen. Schmerz.

[22] Pagotto L.F., Mendlowicz M.V., Coutinho E.S., Figueira I. (2015). The impact of posttraumatic symptoms and comorbid mental disorders on the health-related quality of life in treatment-seeking PTSD patients. Compr. Psychiatry.

[23] Machleidt W., Gül K., Machleidt W., Heinz A. (2010). Kulturelle und transkulturelle Psychotherapie—Tiefenpsychologische Behandlung. Praxis der Interkulturellen Psychiatrie und Psychotherapie.

[24] Norman S.B., Stein M.B., Dimsdale J.E., Hoyt D.B. (2007). Pain in the aftermath of trauma is a risk factor for posttraumatic stress disorder. Psychol. Med..

[25] Kira I.A. (2010). Etiologyand treatment of post-cumulative traumatic stress disorders in different cultures. Traumatology.

[26] Kizilhan J., Utz K.S., Bengel J., Feldmann R.E., Siedler G.H. (2013). Transkulturelle Aspekte bei der Behandlung der Posttraumatischen Belastungsstörung. Traum(a) Migration—Aktuelle Konzepte zur Therapie traumatisierter Flüchtlinge und Folteropfer.

[27] Miranda R., Meyerson L.A., Marx B.P., Tucker P.M. (2002). Civilian-based posttraumatic stress disorder and physical complaints: Evaluation of depression as a mediator. J. Trauma. Stress.

[28] Kirmayer L.J., Young A. (1998). Culture and somatization: Clinical, epidemiological and ethnographic perspectives. Psychosom. Med..

[29] Buhmann C.B. (2014). Traumatized refugees: Morbidity, treatment and predictors of outcome. Dan. Med. J..

[30] Droidek B. (2010). How do we salve our wounds? Intercultural perspectives an individual and collective strategies of making peace with own past. Traumatology.

[31] Slobodin O., De Jong T.J. (2015). Mental health interventions for traumatized asylum seekers and refugees: What do we know about their efficacy?. Int. J. Soc. Psychiatry.

